# Randomized trial comparing low-pressure versus standard-pressure pneumoperitoneum in laparoscopic colectomy: PAROS trial

**DOI:** 10.1186/s13063-020-4140-7

**Published:** 2020-02-22

**Authors:** S. Celarier, S. Monziols, M. O. Francois, V. Assenat, P. Carles, M. Capdepont, C. Fleming, E. Rullier, G. Napolitano, Q. Denost

**Affiliations:** 1 Department of digestive Surgery, Colorectal Unit, Bordeaux University Hospital, 1 Avenue de Magellan, Pessac, 33600 France; 2 Department of Anesthesia ans Critical Care, Bordeaux University Hospital, 1 Avenue de Magellan, Pessac, 33600 France; 3 Department of digestive Surgery, Bordeaux University Hospital, 1 Avenue de Magellan, Pessac, 33600 France

**Keywords:** Laparoscopy, Colectomy, Low-pressure pneumoperitoneum

## Abstract

**Background:**

Laparoscopy, by its minimally invasive nature, has revolutionized digestive and particularly colorectal surgery by decreasing post-operative pain, morbidity, and length of hospital stay. In this trial, we aim to assess whether low pressure in laparoscopic colonic surgery (7 mm Hg instead of 12 mm Hg) could further reduce pain, analgesic consumption, and morbidity, resulting in a shorter hospital stay.

**Methods and analysis:**

The PAROS trial is a phase III, double-blind, randomized controlled trial. We aim to recruit 138 patients undergoing laparoscopic colectomy. Participants will be randomly assigned to either a low-pressure group (7 mm Hg) or a standard-pressure group (12 mm Hg). The primary outcome will be a comparison of length of hospital stay between the two groups. Secondary outcomes will compare post-operative pain, consumption of analgesics, morbidity within 30 days, technical and oncological quality of the surgical procedure, time to passage of flatus and stool, and ambulation. All adverse events will be recorded. Analysis will be performed on an intention-to-treat basis.

**Trial registration:**

This research received the approval from the Committee for the Protection of Persons and was the subject of information to the ANSM. This search is saved in the ID-RCB database under registration number 2018-A03028–47. This research is retrospectively registered January 23, 2019, at http://clinicaltrials.gov/ed under the name “LaPAroscopic Low pRessure cOlorectal Surgery (PAROS)”. This trial is ongoing.

## Background

Laparoscopic colectomies are performed for two types of pathologies: colorectal cancers and benign diseases (Crohn’s disease, ulcerative colitis, diverticulitis, familial adenomatous polyposis, and non-resectable polyps in endoscopy). Currently, laparoscopy is performed for the large majority of digestive surgical procedures instead of laparotomy. The laparoscopic approach for colectomy has better short- and long-term results compared with laparotomy in terms of morbidity, mortality, quality of resection, recurrence, and survival in cancer [1–7]. In the majority of the studies, the authors report a significant decrease in the length of hospital stay in laparoscopic patients. More recently, in a French cohort study of 84,000 patients, it was demonstrated that laparoscopy was the only factor decreasing post-operative mortality [8].

Despite all the apparent advantages of laparoscopy because of its minimal invasiveness (reduction of post-operative pain, improved patient satisfaction, improvement of aesthetic results, and decrease in hospital length of stay), the creation of a pneumoperitoneum has an impact on the cardiovascular and respiratory physiology and has certain limits such as the instability of the pneumoperitoneum, compromised visibility during bleeding, evacuation of smokes, and post-operative shoulder tip pain. Adverse events due to intra-abdominal insufflation of carbon dioxide (CO_2_) are now evaluated, so that their occurrence does not impact the benefits sought by choosing laparoscopic methods [9]. In addition to pain caused by phrenic irritation, residual gas, and abdominal wall stretching, the hemodynamic (reduced venous return and cardiac output and increased peripheral vascular resistance and blood pressure) and metabolic effects of pneumoperitoneum are well-recognized issues [9–14]. From a respiratory standpoint, it reduces lung volume, increases pulmonary resistance, decreases pulmonary compliance, and increases the risk of barotrauma. There is also a possible alteration of renal function. Owing to the absorption of CO_2_, the hypercapnia thus induced can lead to a stimulation of the sympathetic nervous system and increase the plasmatic catecholamines. Owing to these potential effects, European endoscopic guidelines recommend insufflation with the minimum pressure which allows maintenance of sufficient exposure [10]. However, there are no patient outcome data in the literature comparing low- versus standard-pressure pneumoperitoneum in laparoscopic colectomy.

## Objectives

The primary objective is to determine whether low-pressure (7 mm Hg) instead of standard-pressure (12 mm Hg) pneumoperitoneum for patients undergoing laparoscopic colonic resection results in a reduced length of hospital stay of at least 1 day. The secondary objectives are the comparison between groups of post-operative pain, analgesics consumption, 30-day post-operative morbidity, operative time, bleeding, quality of visibility during intervention, conversion rate, perioperative cardiovascular and respiratory function, time to passage of flatus and stool, time to sitting out and mobilizing, and quality of oncological resection (lymph node dissection and curative resection).

## Methods and analysis

### Study design

This is a phase III hospital-based, prospective, randomized, controlled, single-center trial.

### Study population

The patients will be recruited by colorectal surgeons of the Department of Digestive Surgery of the Magellan Medico-Surgical Centre (University Hospital of Bordeaux, France). Patients scheduled for elective colonic laparoscopic surgery for either cancer or benign colonic pathologies (Crohn’s disease, chronic ulcerative colitis, diverticulosis, familial adenomatous polyposis, non-resectable colonic polyps in endoscopy, or other indication) will be assessed for suitability for inclusion.

### Inclusion criteria

Inclusion criteria will be: patients undergoing a right or left colectomy for a malignant or benign pathology, planned laparoscopic procedure, and age of 18 years or older. Following counselling with a member of the research team and provision of written patient information relating to the study prospective, signed informed consent will be obtained from each patient before inclusion.

### Exclusion criteria

Exclusion criteria include: non-laparoscopic procedure, transverse or total colectomy or other procedure performed simultaneously with colonic surgery (except appendectomy or liver biopsy), emergency surgery, surgery for pelvic sepsis, pregnant woman, likely to be or breastfeeding, any patient incapable of providing informed consent, and those unable to commit to the medical follow-up of the study for geographical, social, or psychological reasons.

### Consent and randomization

During the pre-inclusion visit, the investigator will inform the participant and answer all questions regarding the purpose, nature of, foreseeable risks, and the expected benefits of the clinical trial. The investigator is responsible for obtaining informed consent from the participant. An original copy of the information note and signed consent will be given to the patient. The other original copy will be retained by the investigator as part of the study documents in an area inaccessible to third parties. After the written consent of the patient is obtained, randomization will take place the day before or the day of the procedure but at the latest before the incision. When an investigator (“operator” surgeon) wishes to perform the randomization/inclusion after verifying the eligibility of the participant, they will send a randomization application document to the statistician of the study, who will send back a patient number (of three digits corresponding to the inclusion rank), an anonymous letter code (four letters), and the result of the randomization arm, namely group A (experimental): insufflation at low pressure (5–7 mm Hg) or group B (control): insufflation at standard pressure (12–15 mm Hg). The two groups will be balanced with a ratio of 1:1. Randomization will be performed by using the following method: for each subject entering the study, a number K between 0 and 9 will be drawn randomly from Excel. The subject will be assigned to the control group if K is 0 or even or to the “low pressure” group if K is odd. Simple randomization does not necessarily lead to homogenous groups. However, a deviation of less than 20% should have little effect on power loss, and the groups should equilibrate with larger sample sizes (more than 50).

### Blinding

The study will be double-blind. In order to minimize the self-assessment bias of post-operative pain, the patient will not know the group he or she belongs to. The surgeon who decides to discharge the patient from the hospital and who evaluates the primary and secondary endpoints (the “evaluator” surgeon) will not be the “operator” surgeon.

### Intervention

**Fig. 1:**
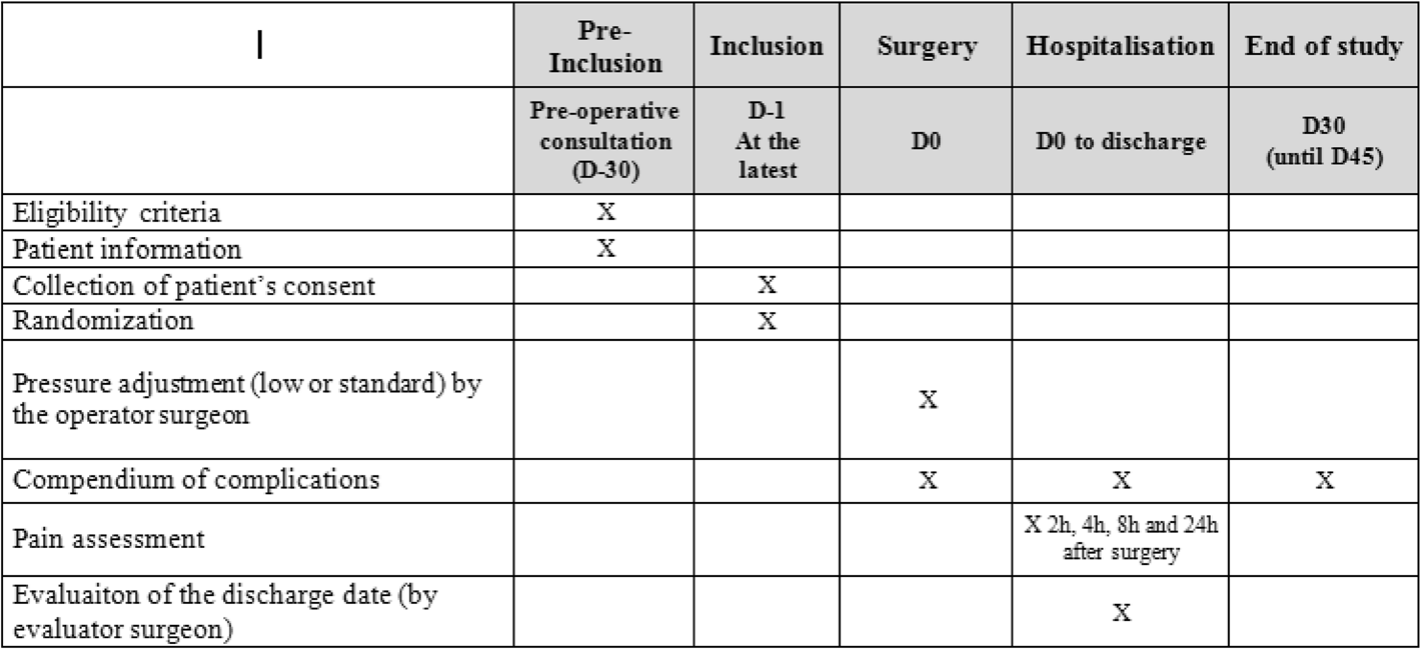
Timeline of PAROS Study

The “operator” surgeon is the surgeon who performs the procedure. The team is made up of six different surgeons, each with their own patients and using the same surgical techniques. The “evaluator” surgeon is the surgeon who validates the patient’s discharge according to predefined and objective criteria. The operating surgeon does not validate the discharge of his own patients. The patient also ignores the arm in which he or she was randomly assigned. The results of randomization are known only by the department’s clinical researchers and kept in a password-protected Excel database. The result is communicated only to the “operator” surgeon on the day of the operation. He knows the randomization arm because it is up to him to adjust the insufflation pressure during the procedure. On day 0, the “operator” surgeon performs a laparoscopic colectomy by using the AirSeal® medical device (ConMed, Utica, NY, USA) and adjusts the insufflation pressure in accordance with the results of the randomization: low-pressure insufflation at 7 mm Hg or standard-pressure insufflation at 12 mm Hg. Different anesthesia and analgesia may affect the secondary outcomes, post-operative pain scores, consumption of analgesics, and recovery time. This is why the anesthetic and analgesic protocol are standardized in both groups and explained in schematic form in the Appendix. The intra-operative protocol starts before the introduction of the trocar: we infiltrate the orifices with 2% naropeine. Then the patient receives a continuous infusion of ultiva, ketamine (0.3 mg/kg as a loading dose and then 0.15 mg/kg per hour until the end of the surgery), and xylocaine (continuous dose of 1.0 mg/kg, stopped 1 h before the end of the surgery). One hour before the end of the surgery, we perform a morphine bolus of 0.1 mg/kg, 50 mg of profenid, and 1000 mg of paracetamol. In the post-operative care room, a morphine titration will be performed if necessary (the amount of morphine given is notified) and the patient receives 1000 mg of paracetamol again 1 h after the surgery is completed. In the post-operative period, the patient systematically receives 1000 mg of paracetamol every 6 h and 50 mg of profenid every 6 h for 48 h. The patient also receives 50 mg of topalgic only if needed and all doses are notified (maximum 8 per 24 h). Perioperative respiratory and cardiovascular management are also standardized. Cardiovascular data will include systolic blood pressure, diastolic blood pressure, mean arterial pressure, heart rate, cardiac index, stroke volume, and stroke volume variation. We also collect the volume of hydration and filling, balance of inputs and outputs, and total doses of ephedrine and noradrenaline (if needed). Respiratory data will include tidal volume, end-tidal CO_2_, respiratory frequency, peak pressure, peak pressure, plateau pressure, driving pressure, compliance (exhalation pressure at 0 and 8 mm Hg), exhalation volume (at 0 and 8 mm Hg), inspired fraction of O_2_, O_2_ saturation, and functional residual capacity. The aerosols are used only if necessary in case of obstructive pathology (asthma or chronic obstructive pulmonary disease). The aerosol used if necessary is Salbutamol (beta-2 mimetics). The degree of muscle relaxation is monitored throughout the interventions using the TOF (Train of Four): four short stimulations of 0.2-ms duration, spread over 2 s. It measures the ratio between the response of the fourth and first stimulation (T4/T1 ratio) or counts the number of responses (from 0 to 4). We measure the TOF via the response of the ulnar nerve (thumb adduction) by using a device located on each patient’s wrist. For all procedures, the TOF must be equal to 0. The post-operative mobilization of the patient and the gastrointestinal recovery will be carried out in accordance with the ERAS (Enhanced Recovery After Surgery) early rehabilitation protocol [15]. Criteria for discharge will be carried out by a surgeon different from the one who performed the operation (“evaluator surgeon”). The patient will be hospitalized for the post-operative period in the colorectal unit of Magellan Medico-Surgical Centre. The follow-up will be standardized regardless of the randomization arm. All inpatient and outpatient adverse events will be recorded and reported. As part of the standard care in our colorectal unit, all patients have a post-operative consultation at 30 days (+15 days maximum) Fig. 1.

### Outcomes

The primary outcome is to determine whether low-pressure (7 mm Hg) instead of standard-pressure (12 mm Hg) pneumoperitoneum during laparoscopic colonic resection leads to a reduction of the length of hospital stay of at least 1 day. The theoretical discharge date is the date of medical discharge of the patient, evaluated daily between 7 and 8 a.m., in accordance with predefined criteria, which are no pain requiring the use of analgesics more than stage 2, no nausea or vomiting, no fever of more than 38 °C, resumption of a normal diet, transit (passage of flatus/stool), patient mobilization, and patient acceptance. The real discharge date is the date of discharge including a possible continuation of hospitalization for non-medical reasons (i.e., waiting for convalescent home, personal suitability of the patient).

Secondary outcomes are to determine how low-pressure pneumoperitoneum (7 mm Hg) influences: patient's post-operative pain (visual analogue scale after 2, 4, 8, and 24 h after surgery), the consumption of level 1, 2, and 3 analgesics, the transit and gas recovery time (days), the time before first time sitting and walking, the evolution of the perioperative cardiovascular and respiratory parameters (arterial blood pressure, heart rate, cardiac output, vascular filling, driving pressure, arterial oxygen saturation [SAO_2_], and post-exercise circulatory occlusion [PECO]), the operating time (from incision to closing, in minutes), the amount of bleeding, the visual quality of the procedure, the laparoscopy conversion rate to normal pressure or laparotomy, ability to achievean R0 resection in cancer surgery, the number of lymph nodes on the specimen and the rate of medical and surgical complications during the first postoperative 30 days (according to the DINDO classification). It seemed important to us to select post-operative pain because it is one of the major factors influencing the patient’s length of stay and very interesting to know whether a lower insufflation pressure makes it possible to reduce pain related to abdominal distention on the one hand and scapular pain on the other hand, which is described as extremely frequent in most laparoscopic procedures. The amount of analgesics (level 1, 2 and 3) is an objective data wich allows us to evaluate pain, wich is a subjective data. In addition, regarding the number of deaths currently associated with chronic opioid use, it seemed that defining whether low pressure allows opioid savings in the post-operative period was a major objective. We also know that taking opioids increases the time before transit resumes and that this influences the length of stay.

### Withdrawal from the study

The participant who wishes to abandon or withdraw consent to participate will no longer be followed in the context of the protocol but will receive medical and surgical follow-up as standard for all post-operative colectomy patients in our unit. Abandonment is a decision of an included participant to assert the right to interrupt participation in a research, at any time during the follow-up, without incurring any prejudice or having to justify the decision. A withdrawal of consent is a decision of a participant to reconsider the decision to participate in this research and to assert the right to cancel informed consent at any time during the follow-up and without incurring any prejudice and without having to justify him- or herself.

### Duration of the trial

Based on 170 colonic laparoscopic resections performed in our unit in 2017, the recruitment of 138 patients in this trial will be performed over 1 year. The duration of participation of each participant will be 2 months, and the total duration of the trial will be 14 months. The inclusion started in January 2019 and was scheduled to finish in January 2020.

### Sample size

The calculation of the number of subjects required is based on the primary endpoint, which is a decrease in hospital length of stay of at least 1 day in patients operated on laparoscopically for malignant or benign colonic pathology using a low-pressure pneumoperitoneum with the medical insufflation device AirSeal®. The activity of the Colorectal Unit of the Magellan Medico-Surgical Centre was 170 colonic laparoscopic resections in 2017, and the average hospital stay of patients in this control arm would be 5 ± 2 days based on available data. As such, in unilateral formulation, to show a difference (1 day) with an alpha risk of 5% and a power of 90%, there should be 69 patients recruited per group, resulting in a total of 138 patients.

### Data management

The data will be collected daily by the investigators. For the intra-operative data (respiratory and cardiovascular components, bleeding, and need for catecholamines or aerosols), they will be collected prospectively and transmitted by the anesthesiologist following each procedure. The exposure will be evaluated just after the procedure by the “operator” surgeon. The visual analogic scale at 2, 4, 8, and 24 h as well as the time before gas, stool, sitting, and walking are reported prospectively on a document that follows the patient at each time. The total amount of analgesics will be calculated on the day of discharge. All information required by the protocol will be recorded on the patient’s study notebook and a second time on electronic notebooks, and an explanation will be provided for each missing data variable. The entry will be made in FileMaker 9.0 version 3 (database software) stored on the network of Bordeaux University Hospital, which guarantees the confidentiality and security of the data processing in accordance with the requirements of the CNIL Reference Methodology. In accordance with the legislation in force, persons having direct access to the source data will take all the necessary precautions to ensure the confidentiality of the data. During or at the end of the research, the data collected on the individuals who are suitable for this analysis will be anonymized prior. The Bordeaux University Hospital network benefits from a daily backup of its data. The database is also stored and archived under the responsibility of the investigator in the colorectal surgery department of the Haut-Leveque Hospital of the Bordeaux University Hospital. For database freezing, a version number and a date will be given to the database, which is kept in a format that no longer allows modifications. A freeze certificate of the database will be completed and sent to the sponsor (EN-RCL-601). Data transfers (sending and receiving) will be carried out in accordance with the CMG procedure. For security reasons, the data files are anonymized and transferred via the secure platform CIRRUS.

### Statistical analysis

The main analysis will focus on all randomly assigned patients (intention-to-treat analysis). The alpha risk is 5% and the power is 90%. Given the main variables studied, there will be no missing data for analysis of the primary outcome. The descriptive statistical analysis will include the following for each quantitative parameter at each time: average, standard deviation, minimum, maximum, median and quartiles, and number of missing values. The categorical variables will be expressed by the frequency of distribution and the associated 95% bilateral confidence intervals. For the primary endpoint, which is theoretical length of hospital stay, the two groups will be compared by using Student’s *t* test if, as suggested by a preliminary study, the distribution of the variable does not deviate significantly from a Gaussian distribution; otherwise, a Mann–Whitney test will be used. For the secondary endpoints, the categorical criteria will be analyzed by using a chi-squared test. In the event that the chi-squared validity criteria are not verified, an exact probability test will be used. Quantitative secondary criteria will be compared by *t* test or Mann–Whitney according to their distribution in the sample considered. All tests will be bilateral at risk of the first species set at 5%. The analysis will be performed by using IBM SPSS Statistics, version 20 software (IBM, Armonk, NY, USA).

### Trial status

The protocol number is ID-RCB: 2018-A03028–47, version n°2.0 from 07/01/2020 promotor code: CHUBX 2018/42. The first inclusion was on January 7 2019; the trial was scheduled to end around Sept 7, 2020. The inclusion period is 18 months with 2 months of participation for the patient.

## Figures and Tables

**Fig. 2:**
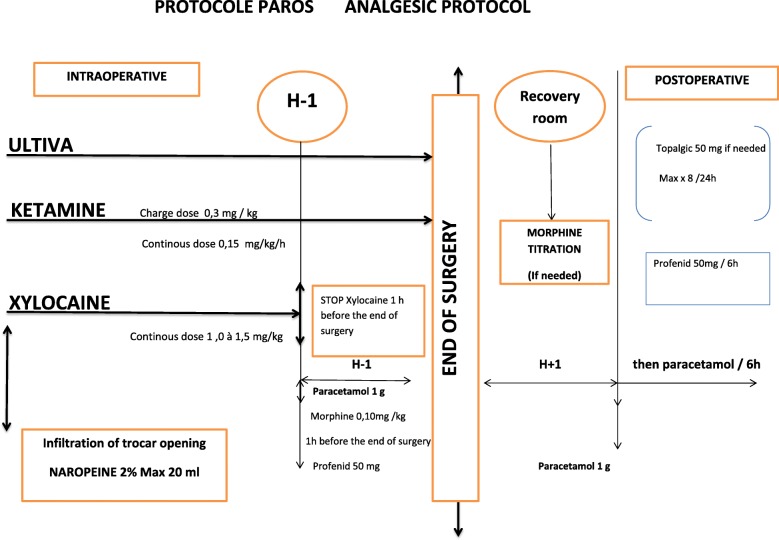
PAROS Study analgesic protocol

## Data Availability

Data analysis will be performed by the principal investigator. This analysis will result in a written report which will be submitted to the sponsor, who will forward it to the Committee for the Protection of Persons and to the competent authority. Any written or oral communication of the results of the research must receive the prior agreement of the coordinating investigator and, where appropriate, of any committee set up for the research. The coordinating investigator undertakes to make available to the public all negative and inconclusive and positive research results. In accordance with the law n ° 2002–303 of March 4, 2002, the participants will be informed, at their request, of the overall results of the research. The results of this study will be published in peer-reviewed journals and be presented at national and international conferences.

## Abbreviations

CO_2_: Carbon dioxide
TOF: Train of Four
